# Determining value of logistics costs in projects: empirical findings based-on executing several cement projects in Indonesia

**DOI:** 10.1016/j.heliyon.2020.e04352

**Published:** 2020-07-15

**Authors:** Effnu Subiyanto, Yohanes Totok Suyoto

**Affiliations:** a Department of Postgraduate School, Faculty of Magister Management, Widya Mandala Surabaya Catholic University, Surabaya, Indonesia; b Department of Management, Universitas Pembangunan Jaya, South Tangerang, Indonesia

**Keywords:** Projects management, Logistics costs, Customs clearance costs, Foreign logistics costs, Domestic manufacture costs, Domestic logistics costs, Insurance costs, Economic development, Pricing, Logistics, Strategic management, Scale construction, Business

## Abstract

This research tries to shed light on precise values of logistics costs that so far undisclosed. The values are important for every project planners to evaluate, control and monitoring, and especially imperative for procurement activities. Tender, bidding, and defining the scope of contracts and value can be well organized without time-consuming. This research uses methodology previously developed by Subiyanto (2020a) and discussed only a few particular and directly related to the logistics. The model had been decomposed by utilizing path analysis and the data had been regressed to define variables as the most inflator for logistics. The findings are total logistics costs in Indonesia generally at around 14.6% to total costs of investment. It consists following of insurance cost 0.11%, costs for customs clearance 6.52%, foreign logistics costs 6.62%, domestic manufacture 0.47%, and domestic logistics costs 0.89%. This research is not discussing logistics costs relating to human resources, human travelling, and internal logistics conducted by vendors or contractors as the scope included counted within their procurement contract. These results are important as guidance for project planners, controllers, and other parties to prepare costs more precisely. Negotiating to the banks will be easier as the more precise amount of budgets had been estimated. Due to the important values that have been identified, there will help transparency and governance conducting the projects. Procurement processes involving societies are more objective and better. This is a novelty finding as previous researchers have not decided yet.

## 1 Introduction

Every project in Indonesia and generally in the world is practically easy to be accomplished, finished, and completed. Starting projects have been marked by groundbreaking ceremony while closing projects would be marked by the closing ceremony. The same couple of ceremonies but at different times with yearly span depend on the size of projects.

While project management has few tasks to make final payments, and gradually part of project teams have been transferred as part of operations’ organization at the new plants; by at the time, the important records, histories, achievements, performances, with its numbers are easily gone and evaporated. Physical documents or electronic data are carefully stored as required as part of the final report for final auditing, but evaluating, assessing or making simply comparison against to previous feasibility study is rarely done.

The most critical thing, while a different team has been assigned to carry other projects –even the same type of project- the new team will face hardly to refer results previous projects achieved as reference for the new projects.

Again, the new team must hire new other consultants, hire several personal experts, rent different tools, and make preparation as like a new team with a new project. Everything has been reset as like a newest and beginning from zero. The amateur-like behavior is totally unproductive and professionalism is highly under questioning.

To disclose numbers; moreover, this case is correlating with contract procurement that is generally not common, unusual, and even more, have been campaigning against the laws. Part of the officers states that the contract is hard to define precise numbers. The numbers will be liquid depends on the market. While supply over the demand will suppress the price, and in contrast, the price will be hike while the demand higher over than supply. This culture-type is retaining advanced information of investment costs and particularly logistics costs that to be addressed in this research.

Based on the situation, this paper had risen. The aim of this paper is to disclose several values associated with project costs particularly in the terms of logistics activities. Unit costs for even the smallest logistics must be carefully identified to make a greater accurate level of results of this research. Due to the findings obtained, these results are a novelty in logistics, giving a contribution to the knowledge and can be a foundation for further research future.

## 2 Literature review

Estimating precisely construction costs at the general physical projects are always becoming a major tasks for every project planners. Ramos (2017) guided a practical in-depth look at the basics to predict construction cost, including how it fits into the construction process and what can cause costs to rise unexpectedly. For more detail, the major types of construction contracts have been presented, best practices, pitfalls for estimators, the use of historical data, and estimation software.

Many subjects have been presented by Ramos (2017) as such proposing nine basic phases in a building project tasks for example (1) determining commissioning team; (2) preparing a pre-design phase; (3) forming a design team; (4) designing the engineering; (5) start of bidding based on the scope of work; (6) signing the contract; (7) construction stages initiate with groundbreaking ceremony; (8) close-out; and (9) completion project.

According to Ramos (2017) from the all-9 basic principle, the most must be concerning is construction stages. Construction then associating with a lot in common with costs must be giving much higher attention therefore for these steps are being structured in several levels. First is the order of magnitude estimate; second is the level of schematic design estimate; third is design development estimate; fourth is the construction document estimate; and the last is bid estimate.

A little bit developed research had been conducted by Bridges (2019) that distributed costs for projects into 6 categories which are (1) direct vs. indirect costs; (2) fixed vs. variable costs; (3) period vs. product costs; (4) pre-operating vs. operating costs; (5) retrospective vs. prospective costs; and (6) opportunity cost vs. the cost of risk.

Marker (2017) joined to study the interestingly topic a bit deeper than Ramos (2017). The study explained an overview of cost estimating, started to disclose key components of a cost estimate. An update of importance using a work breakdown structure (WBS) is incorporating though the technique was not new. Several types of software as like the Critical Path Method (CPM) and the Program Evaluation and Review Technique (PERT) are evaluated to enrich the study. At the level of applied, Marker (2017) is far better than earlier researchers as information technology (IT) had been inserted. The approach undertakes by Marker (2017) to replace modeling using Bayesian Networks approach to help reduce uncertainty project costs presented by Khodakarami and Abdi (2014) and the regression models (Williams, 2003). Furthermore, applying the technique to monitoring projects with control charts to improve the traditional approach is also changed (Aliverdi et al., 2013).

Due to the development of costs in the project that as important as other departments, the procurement department then took bigger portions to contribute. Eriksson (2017) has investigated how procurement strategies must be designed to facilitate exploration and exploitation in construction projects. The collaborative procurement is found in integrating teamwork between contractors and project accounting. The procurement department that can play strategic roles to combine of incentive-based payment, bid evaluation based on multiple criteria and collaborative tools and activities in partnering arrangements and clearly defined scope of the contract (Cheng, 2014).

Even more; good procurement will help to minimize failures of overrun costs of projects that traditionally happen in generally countries (Shehu et al., 2014). The study of Shehu et al. (2014) is likely to respond to the urgent calling of Jorgensen et al. (2012) and Doloi (2011) that prior warning to the need for other types of studies to determine costs projects. However, the strict conditions must be applied that selecting vendors or contractors are based-on fairly evaluation (Rajeh et al., 2015).

Different views with Ramos (2017), Sanchez et al. (2017) measures the importance of organization in the project. The persons that operate organizationally can help minimize uncertainty costs distribution but with robust Information Systems (IS). Sanchez et al. (2017) proposes the Cost and Time Project Management Success (CTPMS) that be able to identify project size, duration, postponement, team size, and team allocation.

However, to precisely determine costs estimation in the projects are remaining mysterious and so far halted by developing model, simulation, and approximately techniques. The earned value management (EVM) method employed from the interval gray numbers (IGNs) has been tested by Nadafi et al. (2019), and also by activity-based costing (ABC) approach (Fang and Ng, 2011). But, there are still questioning to define project costs or building costs as stated by Shehab and Farooq (2013), Alashwal and Chew (2017), and even at large projects (Jayaraman, 2016).

In terms of logistics costs in the projects, not many researchers have studied this particular theme. A Linear Programming (LP) has been performed by Choundhari and Tindwani (2017) at the recent a developing model of logistics costs for projects that had been introduced by Subiyanto (2020a), b. Only a few others have interested to conduct research associated with the cost of trucking (Kotsois and Folinas, 2020) and container transfer (Benghalia et al., 2014).

## 3 Methodology

This study elaborates on the methodology based on empirical cases in the heaviest several cement projects in Indonesia. The path analysis type is introduced and carried out to get a comprehensive mapping of costs, all data then have been tested by the regression method to get results which the highest inflator among other variables that must be appropriately concerned or prioritized.

To convince of the approach; several 8 cement projects were built within period of 2010–2018 have been assessed to get detail results precisely. Data collection had been taken at the whole project during the time construction, be carefully checked and verified daily and sorted in quarterly. Costs associated and incurred to the interest of logistics have been grouped into the category of logistics costs, while costs associated with other interests have been accounted proportionally to other posts.

For data total costs of projects are being differently treated, like nature, due to it accumulated not quarterly separated, then for the case is taken by quarterly accumulated and key-in into the spreadsheet. This technique is to avoid autocorrelation but at the same time keeping data between variables free independently.

**Figure 1:**
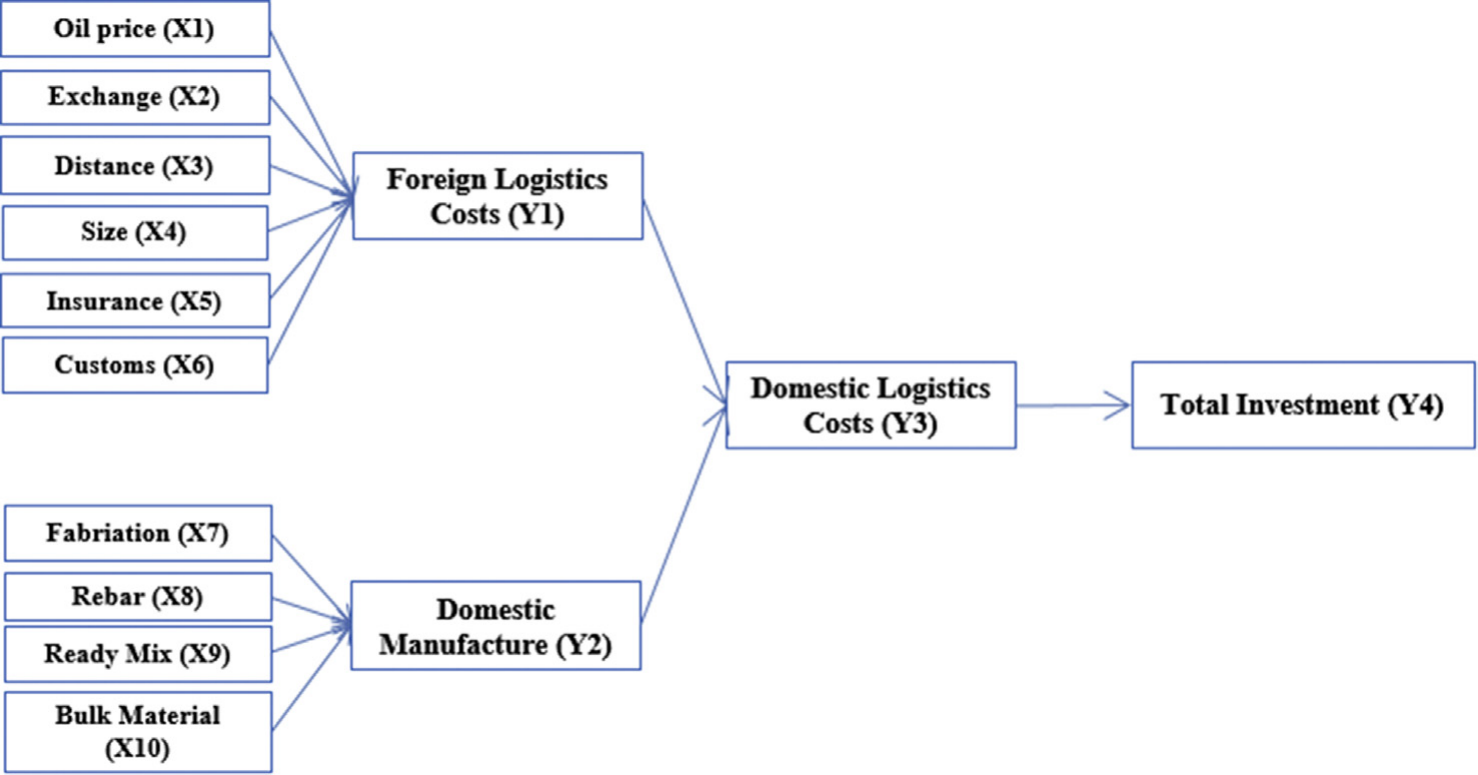
Logistics Model Prior Developed. Source: accommodating from Subiyanto (2020a).

This research utilizing a model developed by Subiyanto (2020a), b as following Figure 1. Subiyanto (2020a), b found the model as revisiting based on literature studies and the model then had been tested by an-EVIEWS10.00 statistical tool to check robustness and weakness. From the point, then it was concluded and decided the model can be adequately popularized to be applied at the general projects, particularly in Indonesia.

Due this research is observing only to discuss associates with the direct logistics costs incurred in every project; therefore, few variables were chosen are (1) insurance; (2) customs clearance; (3) foreign logistics costs; (4) domestic manufacture costs; and (5) domestic logistics costs. To sort of variables considered in this study are of course expert judgements needed. The expert is the only way to decide each factor directly or indirectly associate with logistics.

To calculate logistics costs is by measuring real data taken from the projects. Logistics costs every project has been measured, counted, compared against of the total project costs. The bigger logistics costs is showing more inefficient and vice versa the smallest logistics costs is showing better in organizing logistics costs at the projects.

## 4 Results and discussions

### 4.1 Expert judgement defining relevance variables

**Figure 2:**
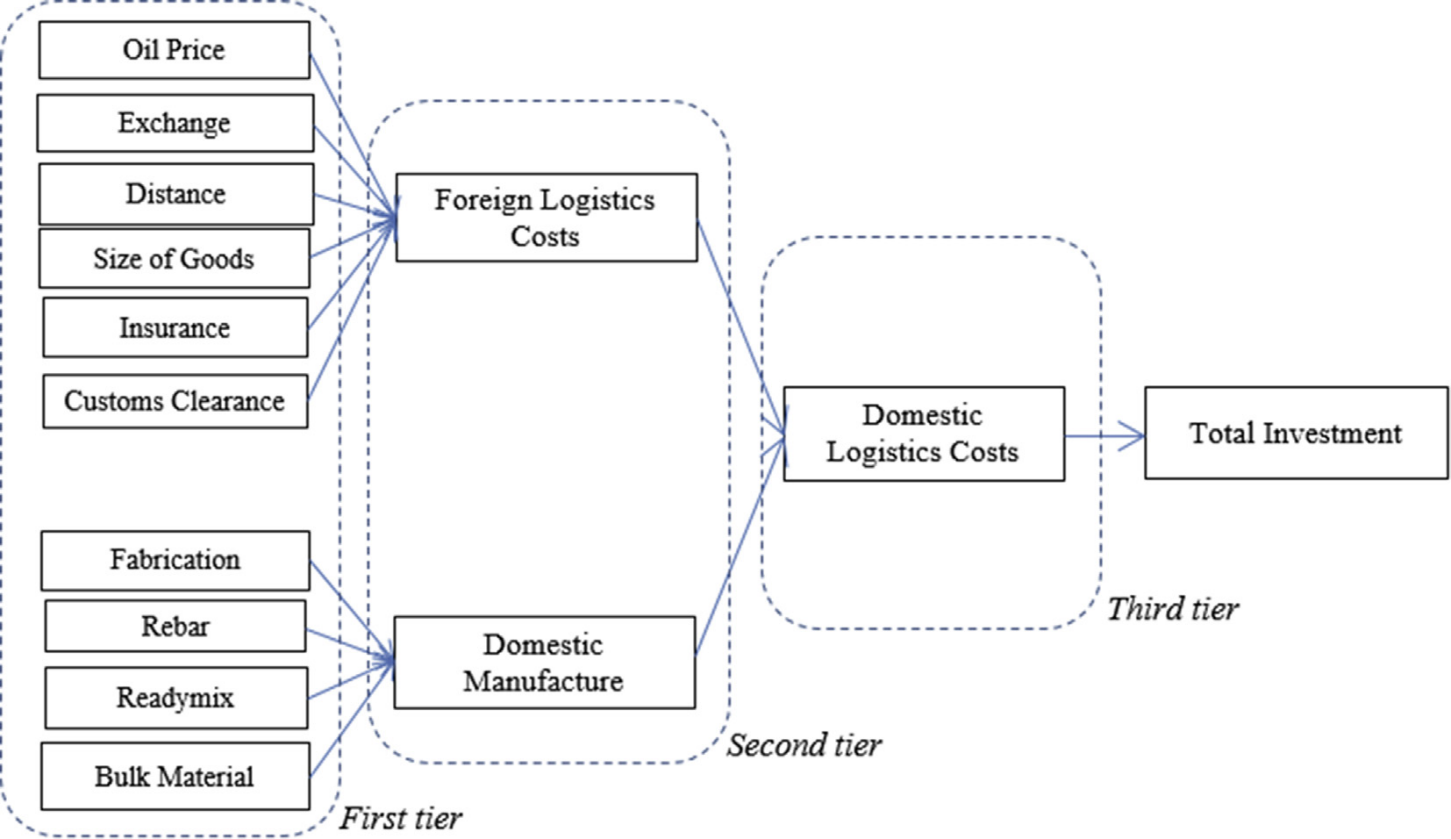
Expert Judgement per Tiers. Source: developed by Author (2020).

To decomposing Figure 1 is by analyzing every tier that built the model. It can be divided into three tiers for simplifying due to the total investment as the dependent variable as a benchmark is not counted. The guidance is following Figure 2. First-tier evaluates all independent variables, second-tier assesses a-first intermediating variables, third-tier considers only a single intermediating domestic logistics costs, and finally a dependent variable of total investment as a primary benchmark of this study.

The tiers are leveling logistics stages identified at the projects. Variables directly related to logistics such as at the portion of foreign logistics is easily identified, but variables correlated came from domestics manufacture must be carefully justified. Domestics manufacturing costs in the case are logistics associated to perform delivery of goods for projects, manufactured origin from the domestics. Movement costs from the originated place to the project site. In the case for example cost delivery of concrete, rebar, plate-works from workshop to project site, bulky materials. This is particular to discuss delivery or logistics costs that generally counted during logistics tenders.

Based-on Figures 1 and 2, the cost of insurance is international mandatory as one of conditional to reserve space in the vessel for delivery. The amount is vary depending on the market price at the time contract. The higher risks then the price insured will be also higher. The goods that are not placed in a container or secured other packages will also be imposed on the higher tariff. The unusual unscheduled type of vessels for example using tramper vessel or specific chartered vessels are also being imposed of higher tariffs of insurance. It is therefore this insurance variable is considering in this study. The expert judgement is also deciding the insurance costs are associated directly as one important factor in the logistics costs.

Besides insurance, costs that directly impacted the projects are costs for customs clearance. This activity is mandatory while dealing with import machinery and common in every country worldwide. The State levied a variety of costs in the form of a variety of taxes or others. In this regard are costs of duty, value-added tax, income tax, and some of them are luxury goods tax. In Indonesia costs of duty are at least 5% to 10%, the value-added tax is 10%, income tax is 2.5% for particular conditionals met, and uncertainty of luxury goods tax to about at least 50% to 500%. This research exempted the luxury goods tax as the costs are not applicable for generally manufacture projects in Indonesia. In this case, the mandatory costs of customs clearance are also taking consideration in this research.

Further, the costs of logistics come from foreign logistics costs. This stage is delivery activities loading and unloading goods for projects generally machinery from point of origin ports to the destination ports in the country where the projects are carried on. The costs are service costs to pick-up machinery from certain places according to procurement contract, loading to the hatch of vessels, sailing on the seas, unloading to the destination ports, and to hand-over goods according agreed the terms based on certain incoterms applied. It is therefore the variable of foreign logistics costs is taking into consideration.

Besides, Subiyanto (2020a), b has decomposed all associates with the delivery of domestic goods within total logistics costs in domestic manufacture. All fabrications have been fitted into the ordinary type of trucks commonly used in Indonesia and being counter-checked with data traffic from the warehouse. The technique has been the same done to treat other variables such as rebar, ready-mix concrete, and bulk material. Total costs associated with domestic manufacture had been tabulated in the variable and therefore this variable is directly impacting the total investment.

Finally, as benchmark primarily studied in this research, the dependent variable of total investment is directly accommodated with a little bit of different treatment. Data on total investment is accumulated based on quarterly; therefore, if we can see tabulation the amount of numbers was increasing but in uncertain multiply. It depends on the amount disbursed according to project progress and correlated with achievement quarterly.

**Figure 3:**
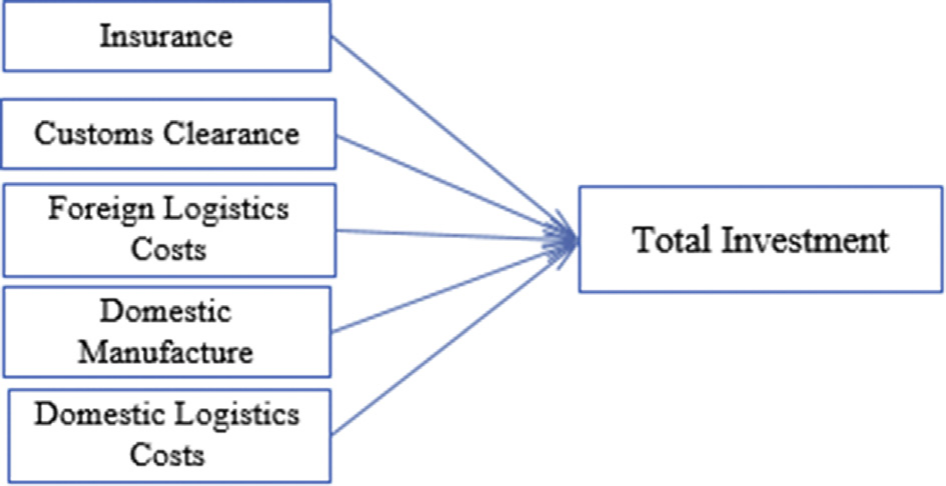
Result of Expert Judgement to Describe Relevance Direct Variables to the Total Investment. Source: developed by Author (2020).

The direct relevance variables of this research then could be defined as following and described by following Figure 3.

### 4.2 Results

Indonesia is a distinctive archipelago-type of country. Geographically, at least 17,000 islands throughout at the country, majorly each of islands separated by water or sea. Since the biggest percentage of total population approximately 272 million people (2019) domicile in Java Island, all disparities then occurred in our today's life. This is not surprising as to make an equal distribution in all islands are requiring prodigious resources. The development model is applied by the current government is building project infrastructure as it mandatory required, not as wishing.

**Figure 4:**
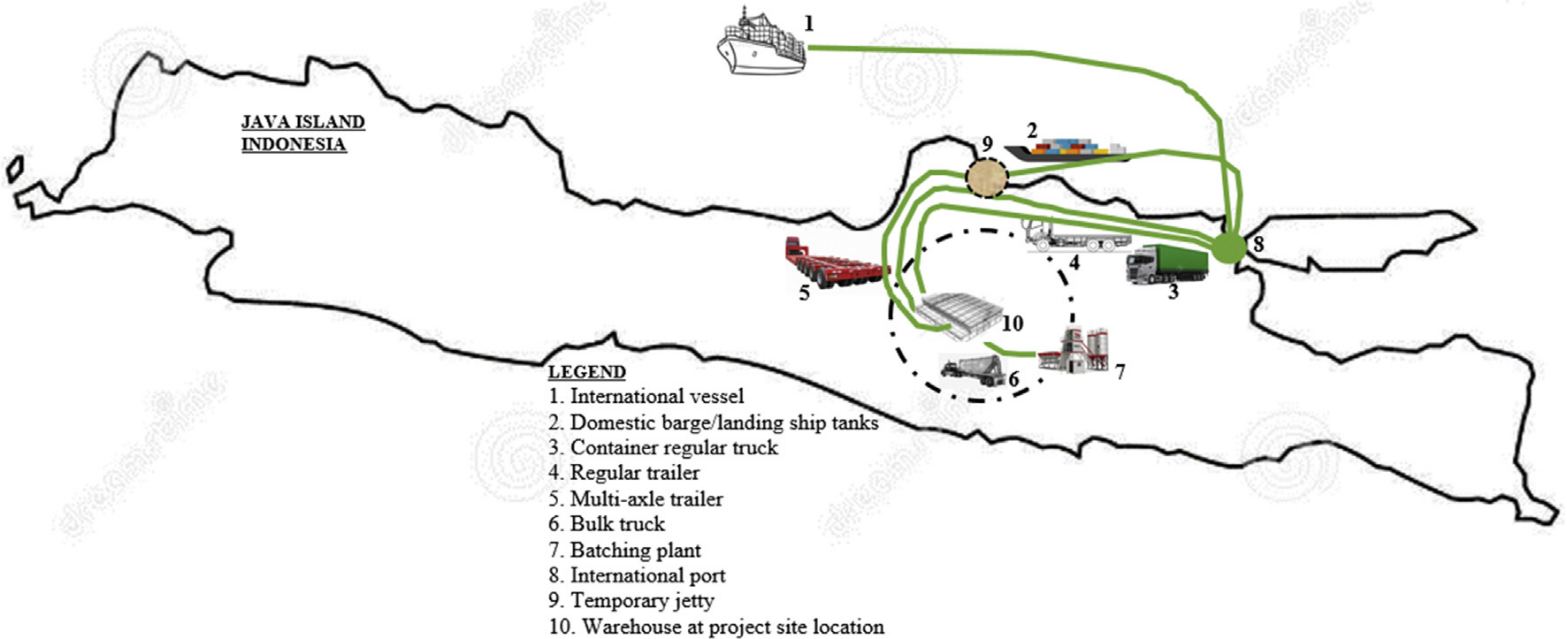
Determining Logistics Route. Source: developed Author (2020).

In terms of executing certain physical projects or cement projects, considering the uncertainty of hurdles or obstacles along the logistics routes; therefore, logistics design must be prior determined. Following Figure 4 to display how to describe logistics routes to executing some cement projects in Java Island. The identical topology is also prevailing to other islands in Indonesia.

Figure 4 is an important step as this design route will be referenced to determine the costs associated with logistics projects. From Figure 4, the international logistics route did not in detail explain as the routes are fixed and certain. But to detailing domestics’ routes are quandary as hundred lines available in Indonesia. Figure 4 explains, there is a domestic sea line from the international sea-port of 8 to the temporary jetty of 9, the nearest to the project site. This path will be serviced by barge-type or small landing ship tank (LST) type of vessel. Applying hydraulic and mechanical jacking as the most practicable instead of using portable cranes that impossible available in a generally remote area. Multi axle-trailer is combining the useful unloading and carrying to the project site.

Few road-type lines must be accommodating by various types of trucks. A route services from certain container-type direct to the project site. A route to service bulky unidentified goods from the center city to the project site, and a route to prepare heavy machines from jetty point to the project site. Within the area of projects itself, there is a single route to carrying concrete from the point of batching plants to the hundred points of constructions or civil foundation.

**Figure 5:**
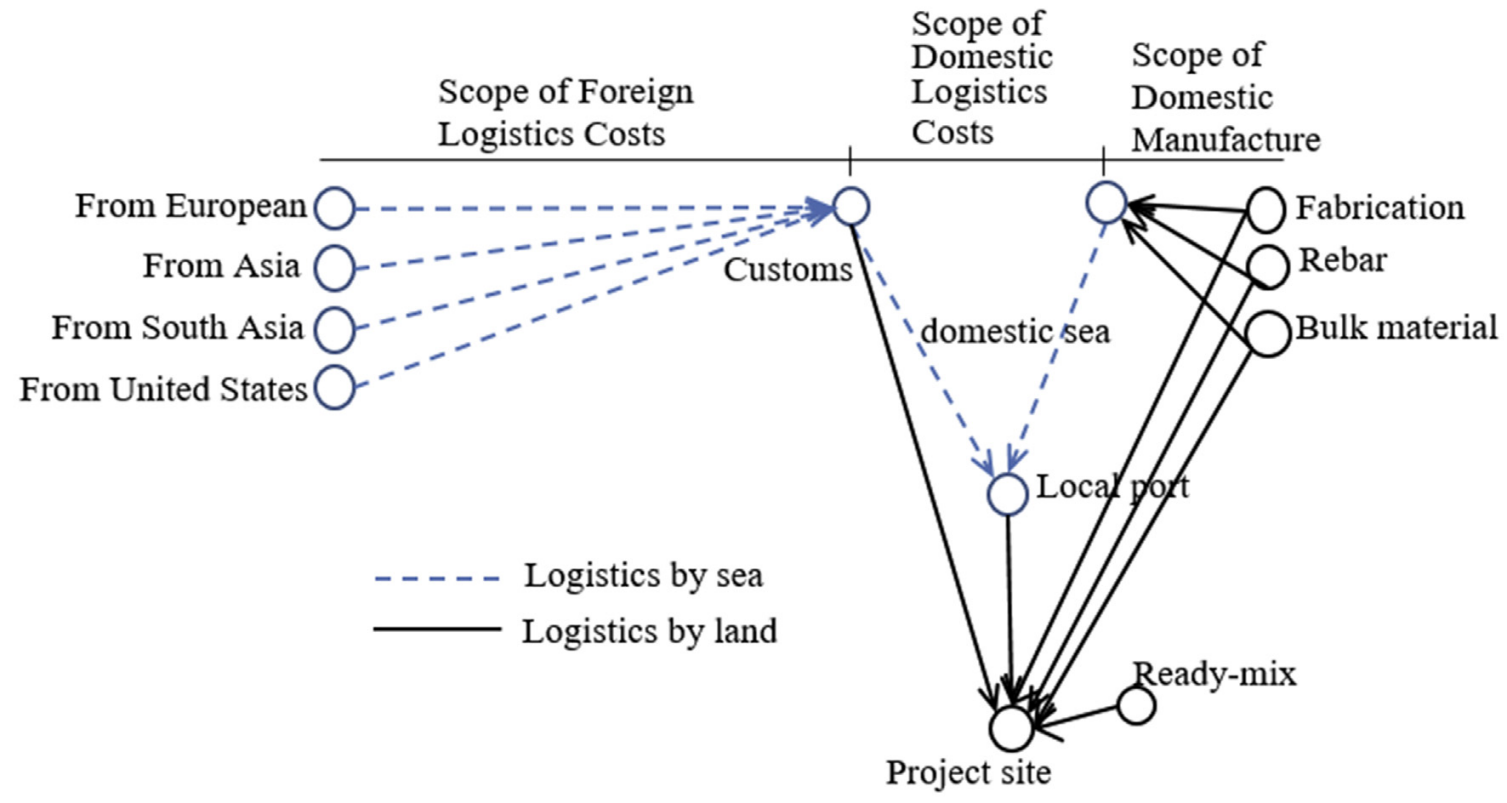
Design Logistics Routes. Source: developed by Author (2020).

From Figures 4 and 5 then developed to help to understand design logistics routes. Figure 5 explains project goods from foreign to be transported by sea to certain Indonesia international seaports. In this section offers two possibilities; goods in containers can be immediately delivered to the project site by regular container trailers, but heavy-lift equipment categories in terms of overweight or over-dimension must be treated to be transported by sea again to the temporary jetty as much as possible at the nearest distance to the project site.

According to Subiyanto (2020a), b based on his experience to study logistics’ route, every line, every route must be precisely drawn to get whole pictures of maps. This is important to describe obstacles and to early anticipate. Bridges of the road especially must be rigorously checked as the heaviest machines could be 220 tons each. The multi axle-trailer type in the case is highly required without be possibly replaced with other types of regular vehicles.

The other obstacles are for example cables that crossing road, banners, billboards, and the most to be early anticipated is the construction of bridges along the roads to the project site. There are two possibilities to strengthen bridges; first by upgrade or make-up construction with additional mechanical supports, or second by using temporary fly-over bridges. The options are both costly, the difference is the first option requires longer construction days while the second option needs a few days as it requires to install and to dismantle.

The costs for uncertainty obstacles in this research have been measured to include in the costs of the domestic manufacture section.

After data had been processed in the template, therefore the real portion of logistics costs at cement projects in Indonesia can be seen in the following Table 1. Total logistics costs in Indonesia based on recent infrastructure real disbursed costs accounted for about 14.60% of total investment costs. The total costs resulted from costs of insurance 0.11%, costs of domestic manufacture 0.47%, costs of domestic logistics costs 0.89%, costs of customs clearance 6.52%, and the highest costs of foreign logistics costs accounted for 6.62%.

**Table 1:** Portion of logistics costs in projects.

Project Name	Insurance	DM	DLC	Customs	FLC	Total
Rembang	0.09%	0.49%	1.73%	5.25%	5.97%	13.53%
Indarung	0.15%	0.54%	0.49%	8.49%	8.66%	18.33%
P14	0.12%	0.60%	0.46%	8.02%	7.97%	17.17%
Tuban#2	0.16%	0.46%	0.39%	9.41%	9.03%	19.45%
Bayah	0.10%	0.37%	1.10%	6.52%	6.34%	14.44%
Banyumas	0.13%	0.45%	1.76%	8.35%	7.87%	18.55%
Maros	0.12%	0.80%	1.30%	7.39%	8.24%	17.85%
Train#1	0.16%	1.00%	0.93%	10.10%	10.37%	22.56%
TOTAL Indonesia	0.11%	0.47%	0.89%	6.52%	6.62%	14.60%

**Figure 6:**
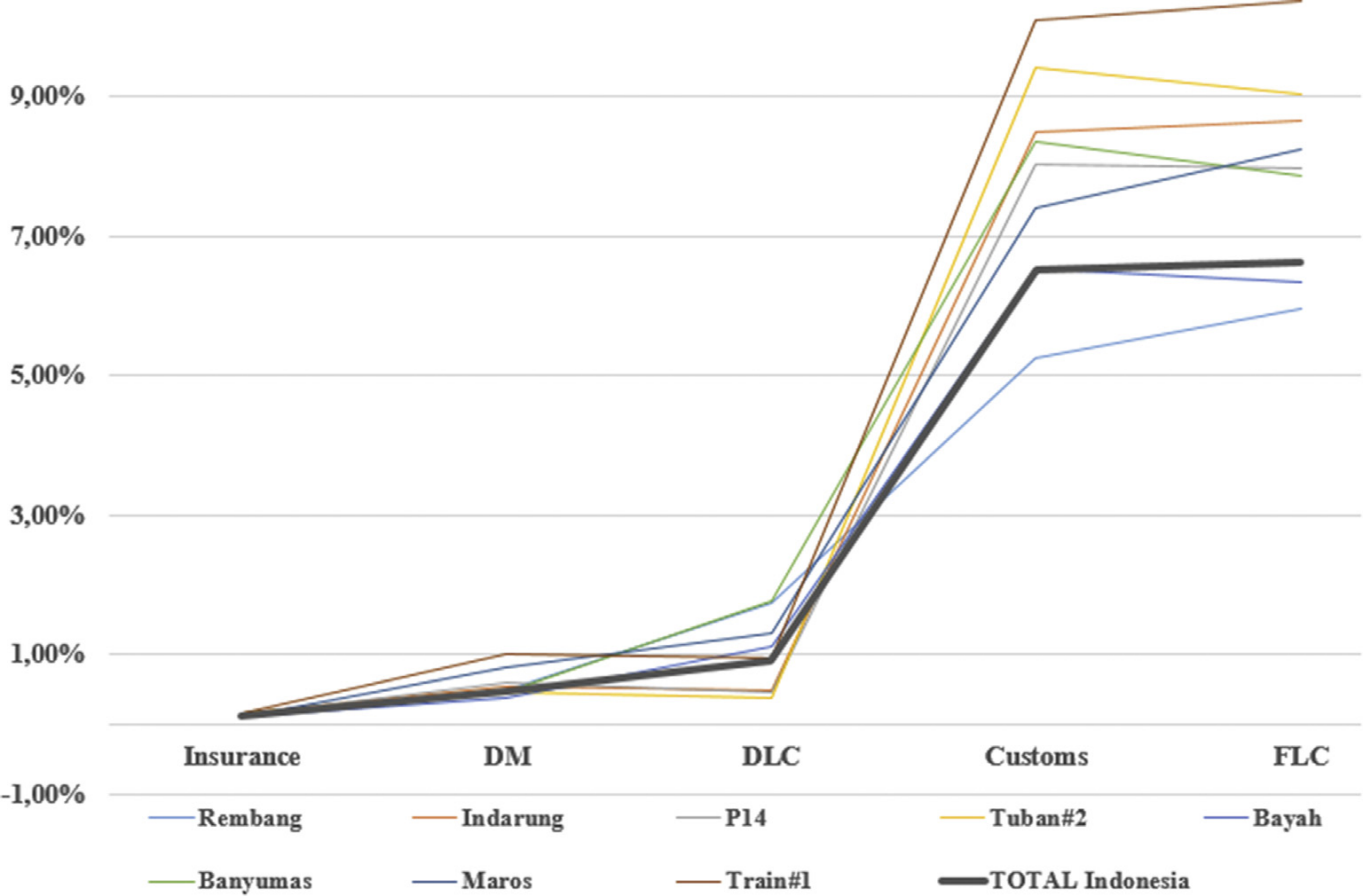
Efficiency comparison between cement projects 2010–2018.

To help easy to make efficiency evaluation between projects based on Table 1 then Figure 6 presents to reveal which total logistics costs of projects above the average line in Indonesia and which projects performed below the line. Projects were carried above the line can be said expensive projects while the others will be said more economical projects. There seem 6 projects above the average while the other two were less than average. The line showing Total Indonesia is the average line in Indonesia.

**Figure 7:**
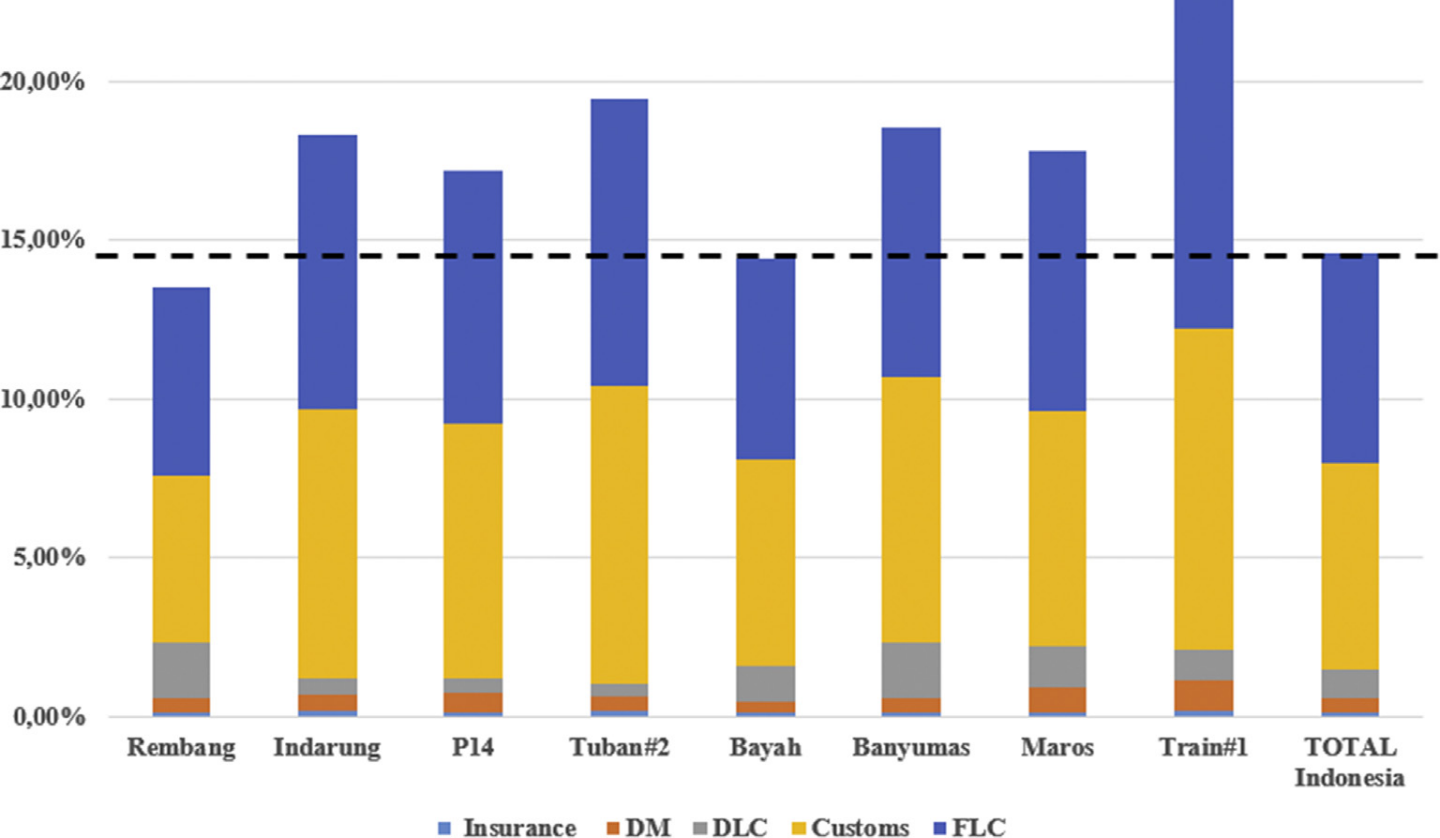
Projects above the national average and below the line.

Easier understanding of graphs can be seen in Figure 7. The logistics projects found above the national average project Train#1 in Kalimantan Island, project Banyumas in Java Island, project P14 in Java Island, project Maros in Sulawesi Island, project Tuban#2 in Java Island, and project Indarung in Sumatera Island. While cement projects found below the line are project Bayah in Java Island, and project Rembang in Java Island.

These findings again proved that better infrastructure is having a substantial contribution to decreasing the total costs of logistics. Java Island particularly has better and much more infrastructure both soft-infrastructure and physical infrastructures to perform all difficult-level of logistics. International seaports in Java Island for example have two, while other islands have none, or at least one. Road infrastructure is much more available in Java Island than in other islands, logistics tools such as vessels, barges, trailers, portable cranes, even more, the multi-axle trailers are also much more ready in terms of quality and quantities. Java Island in general also does not require over than once in multi-modal or transshipment to reach final project site and therefore help decrease the total costs of logistics.

Figure 7 helps to find cement projects that its costs of logistics over the average national-line and below the line. Project Rembang found the lowest costs than 7 others similar projects; even more, the logistics cost the project is below than amount average in Java Island according to Table 2. This is statistical evidence that building a cement plant in Java Island is much more benefitting from the perspective of logistics costs.

**Table 2:** Costs of Logistics based Islands in Indonesia.

Island	Insurance	DM	DLC	Customs	FLC	Total
Jawa	0.12%	0.48%	1.09%	7.51%	7.44%	16.63%
Sumatera	0.15%	0.54%	0.49%	8.49%	8.66%	18.33%
Sulawesi	0.12%	0.80%	1.30%	7.39%	8.24%	17.85%
Kalimantan	0.16%	1.00%	0.93%	10.10%	10.37%	22.56%
TOTAL Indonesia	0.11%	0.47%	0.89%	6.52%	6.62%	14.60%

### 4.3 Regression tests

Costs of logistics in projects based on empirical findings are small, the highest is about 18.33% and the lowest is 16.63% of the total investment costs. It means the major portions of costs will come from other variables than the costs of logistics. The biggest costs within the range of 81.67% to 83.37% must be allocated for procuring machinery, civil construction, fabrication, land clearing, concrete, services, and many more others directly. Due to the reasons by performing regression tests would be no significant impact as the coefficient of adjusted-determination (R^2^) would be absolutely low. This is meaning, the model will be easily interfered with by other variables that outside undefined within the model or unstable model.

However, based on Table 3, it delivers meaning that project Rembang must take care of building better contract logistics costs in handling delivery machinery from the import section. It is therefore variable of foreign logistics costs is predominantly significant.

**Table 3:** Results of regression tests.

Projects	Intercept	Insurance	Customs	FLC	DM	DLC	Adjusted-determinant (R^2^)
Rembang	Not	Not	Not	Significant	Not	Not	0.24713442
Indarung	Significant	Not	Not	Not	Not	Not	-0.0862511
P14	Significant	Not	Not	Not	Significant	Not	0.4008903
Tuban#2	Significant	Not	Not	Not	Significant	Not	0.180002446
Bayah	Not	Significant	Significant	Not	Not	Not	0.516719073
Banyumas	Significant	Not	Not	Not	Not	Significant	0.5305315
Maros	Significant	Significant	Significant	Not	Not	Significant	0.7605823
Train#1	Not	Not	Not	Significant	Not	Significant	0.7642904
TOTAL Indonesia	Not	Not	Not	Not	Not	Not	0.10583073

Project Indarung on the other side as distinctive and specific as none of the regression tests found significant. As the logistics routes in this Sumatera Island, the western part of Indonesia, is more complicated due to demanding several seas multi-modal thus the costs of logistics should be higher than other sides in Indonesia. But none of them significant (as the intercept is not counted) for surely requires further research future. The authors advised for researchers to take part in these findings deliberately.

For project P14 and Tuban#2, both located in Java Island to concern for domestic manufacture. It implies a lot of domestic goods made and therefore demanding accordingly attention. Remain in Java Island, project Bayah located in the most South-western Java Island is dealing with insurance and customs. This project Bayah has a remote area that requires a customs officer at the designed place by its own costs. Therefore the matter of insurance and customs clearance predominantly becomes attention.

Further project Banyumas is having a problem with its project site. As the project at southern Central Java with directly exposing to the Indian Ocean which popular with high-risk delivery of high tide. To anticipate, therefore the project does more utilize roads and therefore costs of domestic logistics variable became significant.

The project of Maros has different scientific arguments. The project site is in Sulawesi Island which an international seaport Soekarno-Hatta exists. Customs clearance can be performed at the Island and therefore a matter of insurance and customs clearance become significant. Besides as the Island is outside Java Island in general limited in logistics infrastructure, it is not surprisingly higher attention to the variable of domestic logistics costs.

Finally, project Train#1 in Kalimantan Island with limited logistics infrastructure and unavailable international seaports. Normally, foreign logistics costs became much more significant along with domestic logistics costs.

However, as this paper mentioned earlier, the total logistics costs amount small number in all costs structure for any projects. Without any statistical tested, every researcher will have immediately answer that the results must be not significant and confirmed according to Table 3. But the important things, this paper has presented and found the empirical-results value that building logistics costs for certain projects. The findings are not limited for the cement projects but it can be popularized to developing in other types of projects.

### 4.4 Discussions

Cement projects are generally identical during preparation, execution, and closing. During preparation, the teams are seeking much more information in almost all aspects. Every information is treated valuable as the information can be referred for further process, bidding, or even more, big tenders. The logistics costs play an important role as logistics is very important to support project execution. Logistics is taking contribution during delivery project goods since the smallest to the biggest, from the base weight unit of a kilogram to the hundred tons.

Learning from several 8 cement projects within the period of 2008–2018 in Indonesia are valuable experiences. Precise data of the projects are gathered, being clustered, for evaluation, and as sources, information to be referred by interested people or others.

The paper can disclose total logistics costs for general projects in Indonesia and could be adopted by other projects. This paper knows how much costs to anticipate insurance, customs clearance, to prepare costs for foreign logistics costs, to reserve costs for handling goods originated from domestic manufactured, and to store adequate costs to perform domestics logistics activities. When the standard has been acknowledged, the smoother processes are going to be easy to be maintained. This is the primary point the use of this paper.

## 5 Conclusion

In real project execution, determining costs to anticipate goods delivery to and from the project site is generally done by a trial-and-error approach. Try at first then immediately applying amendments when the budgets are nearly achieved. These actions are totally false, as outsiders especially project owners will judge the project team not adequately professional. These are common in generally every project execution and the response is always repeating yearly.

To sort all concerning logistics needed for every project, the first to be importantly determined is predicting total goods required for the project. All members of the project team are highly requested to participate in determining the goods needed within each responsibility based on each professional experience. The team starts from the Safety Health Environment (SHE) department, HRD, Engineering, Construction, Procurement, and other organizations within the project, to the Security department.

Based on empirical findings at this research the total logistics costs in Indonesia are at about 14.6% of the total costs of investment. It consists following costs of insurance cost 0.11%, costs for customs clearance 6.52%, foreign logistics costs 6.62%, domestic manufacture 0.47%, and domestic logistics costs 0.89%.

The number findings are particularly important to perform procurement contracts, to negotiate with suppliers or vendors, preparing budgets for the projects, anticipating taxes, valuable for planners and or for project owners, and also for auditors.

Due to the standards is not yet defined clearly to the date, these findings are not excessively said as providing novelty to the discourse related to the nexus between costs designing in every project.

## Declarations

### Author contribution statement

E. Subiyanto and Y. T. Suyoto: Conceived and designed the experiments; Performed the experiments; Analyzed and interpreted the data; Contributed reagents, materials, analysis tools or data; Wrote the paper.

### Funding statement

This research did not receive any specific grant from funding agencies in the public, commercial, or not-for-profit sectors.

### Competing interest statement

The authors declare no conflict of interest.

### Additional information

Supplementary content related to this article has been planned in forthcoming published at International Journal of Applied Logistics (IJAL), ISSN: 1947–9573, 2020, volume 10, issue 2, article 3, titled Assessing Total Logistics Costs: Case Study during the Execution of Cement Projects in Indonesia by author (https://www.igi-global.com/journal/international-journal-applied-logistics/1151).
